# Is the association between infant regulatory problems and trajectories of childhood co-developing internalizing and externalizing symptoms moderated by early screen media exposure?

**DOI:** 10.1007/s00787-024-02634-0

**Published:** 2024-12-31

**Authors:** Ayten Bilgin, Seaneen Sloan, Ross D. Neville

**Affiliations:** 1 Department of Psychology, University of Essex, Colchester, CO4 3SQ UK; 2 School of Education, University College Dublin, Dublin, Ireland; 3 School of Public Health, Physiotherapy and Sports Science, University College Dublin, Dublin, Ireland

**Keywords:** Infant regulatory problems, Internalizing symptoms, Externalizing symptoms, Screen media exposure, Growing Up in Ireland Study

## Abstract

Regulatory problems in infancy are associated with internalizing and externalizing symptom trajectories across childhood, however, it is unknown whether early screen media exposure exacerbates this association. We studied 10,170 individuals from the Growing Up in Ireland ’08 cohort. Parents reported on their children’s regulatory problems at 9 months, screen media exposure at 3 years, and internalizing and externalizing symptoms at 3, 5, 7, and 9 years. Children were categorized based on their patterns of co-developing internalizing and externalizing symptoms. Subsequently, multinominal logistic regression analyses were conducted to estimate whether screen media exposure (> 1 h or > 2 h) moderated the association between regulatory problems in infancy and co-developing internalizing and externalizing symptom classes across childhood. There were four groups of children with distinct patterns of co-developing internalizing and externalizing symptoms. Presence of regulatory problems at 9 months and excessive screen media exposure at 3 years (particularly > 2 h) were independently associated with increased likelihood of all classes. Excessive screen media exposure at 3 years did not significantly moderate the association between regulatory problems at 9 months and internalizing and externalizing symptom trajectories.

**Supplementary Information:**

The online version contains supplementary material available at 10.1007/s00787-024-02634-0.

Emotional and behavioural problems in children frequently co-occur and are characterized by two broad factors: internalizing (i.e., depression and anxiety)and externalizing symptoms (i.e., aggression and hyperactivity) [1]. Internalizing and externalizing symptoms in childhood (particularly if severe and chronic) are linked to adverse outcomes in adolescence and adulthood, such as poor academic achievement and high rates of psychiatric diagnoses [2–4]. Most existing studies on the development of internalizing and externalizing symptoms have examined them separately and at single time-points. However, internalizing and externalizing symptoms in children are not mutually exclusive [5]. Moreover, disaggregating internalizing and externalizing psychopathology fails to recognize and account for the fact that not all children will exhibit the same pattern of internalizing and externalizing symptoms over time. Rather, it is much more plausible that different subgroups of children in the population will exhibit distinct trajectories, characterized by varying levels of internalizing and externalizing symptoms both cross-sectionally and longitudinally [6]. Identifying the early factors that might increase the likelihood of co-developing internalizing and externalizing symptoms in childhood has important clinical applications for practitioners, enabling early identification of children at risk of following distinct trajectories of co-developing internalizing and externalizing symptoms.

## Infant regulatory problems and internalizing and externalizing symptoms

A potential determinant that may distinguish children who are more likely to follow a co-developing internalizing and externalizing symptoms trajectory is infant regulatory problems [7, 8], which include excessive crying beyond 3 months (e.g., difficulties in self-soothing when crying) and sleep problems beyond 6 months of age (e.g., difficulties in settling back to sleep at night) [9]. Infant regulatory problems can occur in isolation (i.e., single regulatory problems) or co-exist (i.e., multiple regulatory problems) as excessive crying and sleep problems are closely related to each other, with one issue potentially exacerbating the other [10, 11]. They are highly-stressful and challenging for parents [9], and are among the most common reasons parents seek help from a medical professional regarding their child’s health during the early years [12].

There is mounting evidence that infant regulatory problems are associated with adverse negative outcomes in later life [8, 13–20]. It has been shown, for example, that regulatory problems as early as 6 months of age increase the likelihood of co-developing internalizing and externalizing symptoms trajectory across childhood [8]. However, limited evidence exists regarding the factors that increase the association between infant regulatory problems and later co-developing internalizing and externalizing symptoms.

### Role of excessive screen media exposure in early childhood

One such factor could be excessive exposure to screen-based media devices (e.g., TV and video viewing) during early childhood. Both the World Health Organization (WHO) and the American Academy of Pediatrics (AAP) have made recommendations about screen time limits for children between 2 and 5 years of age per day (i.e., no more than 1 h and no more than 2 h, respectively) [21, 22] However, it has been widely observed that screen exposure above these recommended guidelines is common [23, 24].

Excessive screen media exposure in early childhood could increase the risk posed by infant regulatory problems, contributing to the development of more severe mental health symptoms in children such as persistent and co-developing internalizing and externalizing symptoms across childhood. The potential interaction between infant regulatory problems and excessive screen media exposure could be explained through two mechanisms. First, for children who struggled with regulating crying and sleeping in infancy, excessive screen media exposure could strengthen patterns of avoidance such as using screens to escape frustration rather than promoting adaptive self-regulation skills. This could be an additional stressor for children who had regulatory problems in infancy, potentially hindering their ability to regulate their emotions and behaviours effectively. This heightened difficulty in self-regulating might intensify their vulnerability, thereby increasing the likelihood of co-developing internalizing and externalizing symptoms trajectories [25]. Second, research indicates that screen media exposure can decrease the duration of parent-infant interactions such as soothing behaviors, verbal encouragement and play, which are essential for buffering the effects of infant regulatory problems [26, 27]. Excessive screen media in early childhood could replace these meaningful interactions, decreasing their protective role and potentially increasing the development of severe internalizing and externalizing symptoms in children who had regulatory problems in infancy.

### The current study

Against this background, the aim of the current study is to investigate whether the association between infant regulatory problems and trajectories of co-developing childhood internalizing and externalizing symptoms differs according to the level of screen media exposure (i.e., > 1 h or > 2 h per day) in early childhood. We hypothesized that both infant regulatory problems and excessive screen media exposure in early childhood will predict internalizing and externalizing symptom trajectories with larger effects for screen media exposure > 2 h per day. We also hypothesized that the association between infant regulatory problems and internalizing and externalizing symptom trajectories will be larger for children who had excessive screen media exposure in early childhood, with larger effects for screen media exposure > 2 h per day. To identify developmental subtypes of internalizing and externalizing symptom trajectories, while considering that these symptoms can co-occur, the current study applied parallel process latent class growth analysis (PP-LCGA) modelling [8, 28–30].

## Methods

### Participants

The current study used data from the Growing Up in Ireland (GUI) Infant Cohort ‘08, which is a nationally representative sample of 11,194 children born in Ireland between December 1, 2007, and June 30, 2008. The GUI project was administered by trained Officers of Statistics from the Irish Central Statistics Office, and recruitment and data collection were carried out using face-to-face household interviews in a manner akin to the approach used for the Census of the Population. Ethical approval for the project was overseen by the Irish Department of Children and Youth Affairs. Caregivers provided informed consent and received no financial incentive for study participation. Families were recruited when the child was 9 months old and followed up thereafter at two-year intervals. The constructs of interest for this study are from waves one to five (i.e., 9 months, 3, 5, 7, and 9 years). Detailed information on the sampling and scope of GUI is available at: https://www.growingup.gov.ie/.

At 9 months, 11,134 participants completed the survey. The final sample for this study included 10,170 participants who had at least one internalizing and externalizing symptom measure in childhood (waves 2–5) (91.3% of the original sample at 9 months). To assess whether loss to follow-up had been random or selective, those who dropped out (*N* = 964) were compared to those who were retained in the study (*N* = 10,170). Those lost to follow up and those who retained in the study were similar in terms of the percentages of female (*N* = 451, 46.8% vs. *N* = 5004, 49.2%, *p* = .151). However, those lost to follow up were more often from a non-Irish background (*N* = 397, 41.7% vs. *N* = 1883, 18.6%, *p* <.001), and had the lowest quintile of annual income (*N* = 297, 34.9% vs. *N* = 1922, 20.4%, *p* <.001) than those retained in the study.

### Measures

**Internalizing and externalizing symptoms across childhood**. Internalizing and externalizing symptoms were measured using the Strengths and Difficulties Questionnaire (SDQ), which was completed by parents at four assessment points when children were 3, 5, 7 and 9 years. The SDQ is a widely used and psychometrically valid behavioral screening tool suitable for community samples [31]. In line with recommendations [32], internalizing symptoms were assessed by combining the 5-item negative emotionality (e.g., ‘child has many worries’) and the 5-item peer problems (e.g., ‘often fights with other children or bullies them’) sub-scales. Scores for each of the 10 items (0 = not true, 1 = somewhat true, 2 = certainly true) were added to derive a total score ranging from 0 to 20. Externalizing symptoms were assessed by combining the 5-item conduct problems (e.g., ‘child often cheats or lies’) and the 5-item hyperactivity (e.g., ‘child is easily distracted’) sub-scales, scored in the same way to derive a total score ranging from 0 to 20.

**Regulatory problems at 9 months**. Parents reported on their infants’ crying problems using the following question: ‘*Do you feel that baby’s crying is a problem for you?*’ The response scale (1 = yes; 2 = no) was recoded to reflect a crying problem (0 = no; 1 = yes). Parents reported on sleeping problems of their infants using the following questions: (a) ‘*Is your baby ever difficult when put to bed?*’ The original response scale (1 = most of the time; 5 = never) was recoded to a dichotomous response reflecting the presence of a sleeping problem (0 = never, often, at times, rarely; 1 = most of the time); (b) ‘*How often does your baby wake at night?*’ The response scale (1 = never; 5 = more than once per night) was recoded to reflect a sleeping problem (0 = never, occasionally, most nights, every night; 1 = more than once per night); (c) ‘*How much is baby’s sleeping pattern or habits a problem for you*?’ The response scale (1 = a large problem; 4 = no problem at all) was recoded to reflect a sleeping problem (0 = no problem at all, small problem, moderate problem; 1 = a large problem). The Cronbach alpha of these three items reflecting sleeping problems was 0.81. For sleeping problems, these three questions were summed, and dichotomised (0 = no sleep problems; 1 = 1, 2, or 3 sleep problems). Afterwards, crying and sleeping problems scores were summed to create a regulatory problems score: 0 = no regulatory problems, 1 = single regulatory problems (either crying or sleeping); 2 = multiple regulatory problems (co-occurrence of crying of sleeping).

**Excessive screen media exposure at 3 years**. Caregivers reported the time children spent watching television and using electronic devices when the children were 3 years of age with the following prompt: ‘*Typically*, * how many hours a day does < child name > sit and watch television or videos/dvds*?’. Screen exposure time was reported by caregivers in minutes and was categorized as follows: a) > 1 h per day (0: <= 60 min; 1: >60 min) based on WHO guidelines [21]; and b) > 2 h per day (0: <= 120 min; 1: >120 min) based on AAP guidelines [22].

**Covariates**. The following demographic variables were used as covariates: child sex at birth (0 = male, 1 = female), equivalized annual income quintiles (1 = lowest; 2 = 2nd; 3 = 3rd; 4 = 4th; 5 = highest), majority ethnicity: parent-reported ethnic status (0 = non-Irish including any other White background, African or any other Black background, Chinese or any other Asian background, Other including mixed background; 1 = Irish), marital status at 9 months (0 = single parenting; 1 = cohabiting, married), and second or later born (0 = first born, 1 = second or later born) entered as categorical variables, and gestational age (weeks) and age of the main carer at birth as continuous variables. Further, maternal psychological distress was assessed at 9 months using the Kessler Psychological Distress Scale [33], a widely used brief screening tool for mental health problems in the general population. It includes 6 items (e.g., ‘*How often did you feel hopeless?*’) rated on a 5-point scale ranging from ‘none’ to ‘all of the time’ that assess psychological distress in the past 30 days. A mean value was computed, with higher scores reflecting higher symptoms of mental health problems.

### Statistical analysis

Descriptive statistics were used to summarize the characteristics of the sample. To estimate the differences between infants with and without any regulatory problems at 9 months of age in terms of outcome variables (i.e., internalizing and externalizing symptoms), covariates, and the potential moderator (i.e., screen media exposure), we performed independent samples t-tests for continuous variables and chi-square test for categorical variables using SPSS 27 (IBM Corp., Armonk, NY, USA). To address our main hypothesis, we followed a two stage process [8] as described in Jung and Wickrama (34). To address missing data, we used a maximum likelihood estimator and missing data were replaced using the full information maximum likelihood procedure.

#### Stage one

In stage one, we conducted parallel process latent class growth analysis (PP-LCGA) [30] to identify distinct groups of children exhibiting similar longitudinal patterns of co-developing internalizing and externalizing symptoms. This analysis was performed using MPlus (version 8, Muthen & Muthen, Los Angeles, CA, USA). PP-LCGA is a special case of growth mixture modeling that assumes homogeneity of growth parameters within each latent subgroup. Consequently, it can discern homogenous classes defined by different developmental trajectories of co-developing symptoms. The full information maximum likelihood approach was used to impute missing data.

The child’s sex at birth was incorporated as a covariate in the PP-LCGA due to previous evidence indicating sex differences in the development of internalizing and externalizing symptoms [29], which helped to avoid misspecification of subjects into latent classes [34]. We used the automatic R3STEP approach to model sex as an auxiliary variable, which adjusts for the impact of covariates while estimating the number of latent classes. This approach has been shown to produce less-biased estimates than traditional methods [35]. Other confounding variables were not included at this stage due to the considerable computational intensity required to estimate PP-LCGA models.

To determine the optimal number of latent classes, we examined several model fit indices, including Bayesian information criterion (BIC) and Akaike information criterion (AIC), Lo–Mendell–Rubin (LMR), Vuong–Lo–Mendell–Rubin (VLMR), and the entropy value [34]. Briefly, we estimated one to six classes and selected the best fitting model based on fit indicators. In addition to statistical model fit indices, several other criteria were considered to determine the optimal number of latent classes, including ensuring the smallest class should include at least 5% of the sample, the parsimony of models, their interpretability, and theoretical justification [17, 29].

#### Stage two

For stage two, we derived latent classes, saved them in a data file, and imported them into SPSS 27 for further analysis. We used the imported internalizing/externalizing classes to examine associations with predictors and internalizing/externalizing symptom classes across childhood. We conducted multinominal logistic regression analysis following the procedure outlined by Aiken and West [36]. This involved three steps: (a) including covariates (i.e., female sex, ethnicity, maternal psychological distress, income, gestational age, parity, marital status) and regulatory problems (i.e., single, multiple); (b) including excessive screen media exposure in early childhood (‘<=1 hour vs. > 1 hour’, and ‘<= 2 hours vs. > 2 hours’); and c) incorporating interaction terms between regulatory problems and the two levels of excessive screen media exposure.

#### Interpretation

We estimated the odds of membership in each latent class at different levels of our independent variable (infant regulatory problems) and moderating variable (excessive screen media exposure). Regarding regulatory problems, the OR represents the odds of class membership when an infant has any regulatory problems (i.e., either single or multiple regulatory problems) in comparison to not having regulatory problems. Regarding excessive screen media exposure, the OR represents the odds of class membership when a child was exposed to excessive screen media in comparison to no excessive screen media exposure. Given the likelihood of finding significant *P* values < 0.05 for small effects when using large sample sizes (*N* > 10,000), the results are interpreted both in terms of their magnitude (i.e., effect size) and respective 95% confidence intervals (CIs) [24].

#### Further analysis

To facilitate a clearer interpretation of the results, we additionally calculated the unadjusted and adjusted odds of class membership using children with ‘regulatory problems at 9 months and no excessive screen media exposure at 3 years’ as the reference group relative to ‘children with regulatory problems at 9 months and excessive screen media exposure at 3 years (separately for more than 1 or 2 hours of exposure)’.

## Results

The prevalence rate of any regulatory problems at 9 months was 22.4%. Most infants had a single regulatory problem (*N* = 2000; 19.7%), while a smaller percentage had multiple regulatory problems (*N* = 280; 2.8%). On average, daily screen time at 3 years was 109.46 min (SD = 67.98). The percentage of exposure to screen media for > 1 h was 61.6%, and > 2 h was 25.1%. A larger proportion of infants with any regulatory problems were male, from minority ethnicities, exposed to more excessive screen media at 3 years, and exhibited higher internalizing and externalizing symptoms from 3 to 9 years of age in comparison to infants who did not have regulatory problems at 9 months (Table 1). Parents of infants with any regulatory problems were more likely to have the lowest quintile income, higher scores in psychological distress, and be single parents. There were no other differences between children with and without regulatory problems. Bivariate correlations are shown in Supplementary Table 1.

**Table 1:** Descriptives of the sample

	Regulatory problems (*N* = 2280; 22.4%)	No regulatory problems (*N* = 7877; 77.6%)	*p*
Infant sex			0.04
Female: *N* (%)	1079 (47.3%)	3920 (49.8%)	
Male: *N* (%)	1201 (52.7%)	3957 (50.2%)	
Gestational age (weeks): M (SD)	39.51 (1.96)	39.48 (2.11)	0.55
Birth weight (grams): M (SD)	3491.63 (528.74)	3485.62 (537.02)	0.63
Parental age (years): M (SD)	31.84 (5.34)	31.77 (5.24)	0.58
Ethnicity: *N* (%)			< 0.001
Irish	1707 (75.1%)	6548 (83.4%)	
Non-Irish	567 (24.9%)	1305 (16.6%)	
Income: *N* (%)			< 0.001
Lowest quintile	507 (24.1%)	1412 (19.3%)	
2nd quintile	436 (20.7%)	1303 (17.8%)	
3rd quintile	393 (18.7%)	1438 (19.7%)	
4th quintile	420 (19.9%)	1655 (22.7%)	
Highest quintile	350 (16.6%)	1497 (20.5%)	
Marital status: *N* (%)			0.006
Single parenting	745 (33.1%)	2349 (30.1%)	
Married	1503 (66.9%)	5451 (69.9%)	
Parity: *N* (%)			0.064
First born	865 (37.9%)	3158 (40.1%)	
Second or later born	1415 (62.1%)	4719 (59.9%)	
Maternal depression: M (SD)	3.24 (4.12)	2.16 (3.34)	< 0.001
Screen media exposure: *N* (%)			
>1 h per day	1390 (63.6%)	4623 (61%)	0.029
>2 h per day	593 (27.1%)	1861 (24.5%)	0.015
INT at 3 years: M (SD)	2.80 (2.33)	2.41 (2.14)	< 0.001
INT at 5 years: M (SD)	2.76 (2.56)	2.38 (2.34)	< 0.001
INT at 7 years: M (SD)	3.19 (3.18)	2.82 (2.88)	< 0.001
INT at 9 years: M (SD)	3.37 (3.12)	2.86 (2.83)	< 0.001
EXT at 3 years: M (SD)	5.71 (3.54)	5.16 (3.26)	< 0.001
EXT at 5 years: M (SD)	5.06 (2.54)	4.61 (2.38)	< 0.001
EXT at 7 years: M (SD)	4.51 (3.43)	4.12 (3.24)	< 0.001
EXT at 9 years: M (SD)	4.44 (3.52)	4.11 (3.39)	< 0.001

INT: internalizing symptoms; EXT: externalizing symptoms; p: *p*-value; M: mean; SD: standard deviation

### Parallel process latent class growth analysis: co-developing internalizing and externalizing symptom classes

Table 2 displays the fit indices for each model. AIC and BIC decreased with the addition of each class indicating a better fit for more classes. However, decreases in AIC and BIC became considerably smaller from 4 classes onwards. Although both VLMR and LMR indicated a significant improvement in the model fit for 5 classes (*p* < .01), we selected the 4-class model since the entropy value became smaller in the 5-class solution and the 5-class solution included a class with less than 5% of the sample.

**Table 2:** Model fit indices of Parallel Process Latent Class Growth Analysis (PP-LCGA)

Number of Classes	Bayesian Information Criteria (BIC)	Akaike Information Criteria (AIC)	Lo-Mendell-Rubin (LMR) *p* value	Vuong-Lo-Mendell-Rubin (VLMR) *p* value	Parametric Bootstrapped Likelihood Ratio Test *p* value	Entropy	Number of subjects per class
1	319678.936	319592.209					
2	306496.862	306373.999	< 0.001	< 0.001	< 0.001	0.824	2376/7794
3	303089.683	302930.685	0.006	0.006	< 0.001	0.773	750/6234/3186
4	300293.977	300489.112	< 0.001	< 0.001	< 0.001	0.789	1263/642/2029/6236
5	299145.989	298914.718	0.012	0.011	< 0.001	0.750	2474/1214/840/444/5198
6	298269.090	298001.683	0.406	0.400	< 0.001	0.760	381/5150/554/732/2419/934

**Fig. 1:**
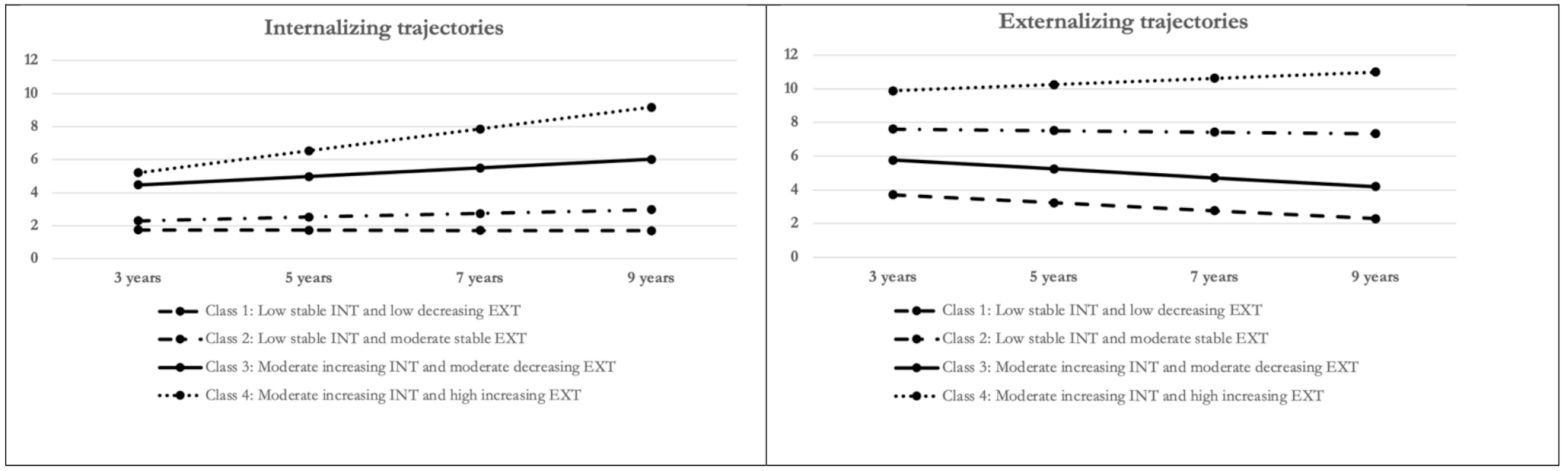
Parallel process latent class growth analysis of co-developing internalizing and externalizing symptoms trajectories

**Fig. 2:**
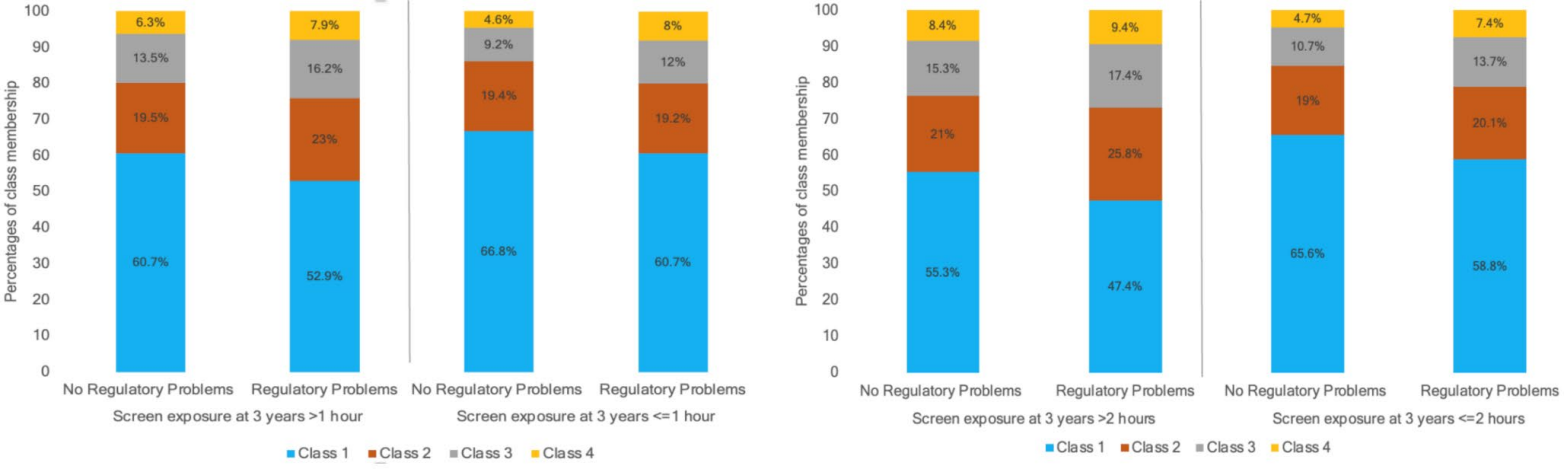
Percentages of class membership categorized by regulatory problems at 9 months and excessive screen media exposure at 3 years

Figure 1 displays the four classes. Class 1, the largest, comprised 61.3% of the sample and was characterised by low stable internalizing and low decreasing externalizing symptoms. Class 2, the second largest, included 20% of the sample and was characterised by low stable internalizing and moderate stable externalizing symptoms. Class 3 consisted of 12.4% of the sample and was characterised by moderate increasing internalizing and moderate decreasing externalizing symptoms. Class 4, the smallest, contained 6.3% of the sample and was characterised by moderate increasing internalizing and high increasing externalizing symptoms. Classes 1, 2, and 3 contained similar percentages of males and females, while class 4 had a higher percentage of males (12.9%) compared to females (6.3%). Figure 2 depicts the percentages of class membership categorized by regulatory problems at 9 months and the level of excessive screen media exposure at 3 years.

### Associations between regulatory problems at 9 months, excessive screen media exposure at 3 years, and co-developing internalizing and externalizing symptom classes from 3 to 9 years

Regulatory problems at 9 months of age were associated with each childhood internalizing and externalizing symptom class (using class 1 as the reference group; Table 3). For instance, regulatory problems at 9 months were associated with increased likelihood of belonging to class 2 (OR = 1.15; 95% CI = 1.03–1.28), which slightly increased for class 3 (OR = 1.21; 95% CI = 1.06–1.37) and increased more for the rate of belonging to class 4 (OR = 1.36; 95% CI = 1.16–1.60). The magnitude of these odds ratios and their 95% CIs were mostly unaffected when excessive screen media exposure at 3 years was taken into consideration.

**Table 3:** Adjusted multinomial logistic regressions between regulatory problems at 9 months, excessive screen media exposure at 3 years (more than 2 h), and internalizing and externalizing (INT/EXT) symptom classes (3 to 9 years)

Steps			OR (95% CI)
			Adjusted^a^
1	Regulatory Problems	Class 1	[reference]
		Class 2	**1.15 (1.03–1.28)**
		Class 3	**1.21 (1.06–1.37)**
		Class 4	**1.36 (1.16–1.60)**
2	Regulatory Problems	Class 1	[reference]
		Class 2	**1.15 (1.03–1.29)**
		Class 3	**1.19 (1.04–1.35)**
		Class 4	**1.33 (1.12–1.57)**
	Screen media exposure more than 1 h	Class 1	[reference]
		Class 2	0.95 (0.83-1.07)
		Class 3	**1.29 (1.10–1.51)**
		Class 4	0.88 (0.71 − 1.10)
	Screen media exposure more than 2 h	Class 1	[reference]
		Class 2	**1.26 (1.09–1.46)**
		Class 3	**1.32 (1.12–1.55)**
		Class 4	**1.71 (1.36–2.15)**
3	Regulatory problems X Screen media exposure more than 1 h	Class 1	[reference]
		Class 2	1.16 (0.90-1.51)
		Class 3	0.97 (0.71-1.31)
		Class 4	0.63 (0.45- 0.87)
	Regulatory problems X Screen media exposure more than 2 h	Class 1	0.79 (0.53-1.18)
		Class 2	1.01 (0.77-1.34)
		Class 3	0.88 (0.64 − 1.20)
		Class 4	0.91 (0.60-1.38)

^a^Adjusted for female sex, ethnicity, maternal psychological distress, income, gestational age, parity, marital status

Boldface type indicates significant associations

Class 1 (Low stable INT and low decreasing EXT); Class 2 (Low stable INT and moderate stable EXT); Class 3 (Moderate increasing INT and moderate decreasing EXT); Class 4 (Moderate increasing INT and high increasing EXT)

Screen media exposure for > 1 h was associated with increased likelihood of class 3 membership (OR = 1.29; 95% CI = 1.10–1.51), but was not associated with the odds of belonging to classes 2 and 4. Screen media exposure for > 2 h was associated with increased likelihood of membership in each classes, with a linear increase in odds ratios of 1.26 (95% CI = 1.09–1.46) for class 2, 1.32 (95% CI = 1.12–1.55) for class 3, and 1.71 (95% CI = 1.36–2.15) for class 4.

Excessive screen media exposure at 3 years of either > 1 h or > 2 h did not moderate the association between regulatory problems at 9 months and the likelihood of membership in the different classes of internalizing and externalizing symptom trajectories.

When we estimated the unadjusted odds of class membership using children with ‘regulatory problems at 9 months and no excessive screen media exposure at 3 years’ as the reference group, children with ‘regulatory problems at 9 months and excessive screen media exposure at 3 years (either more than 1 or 2 hours) had increased likelihood of being in classes 2 and 3. However, this association disappeared after adjusting for covariates (Supplementary Table 2). After adjusting for covariates, children who had regulatory problems at 9 months and excessive screen media exposure at 3 years of more than 2 hours were less likely to be in class 1 in comparison to children who had regulatory problems at 9 months and no excessive screen media exposure at 3 years (OR = .75; 95% CI = .59-.96).

## Discussion

A large community sample was used to examine whether excessive screen media exposure in early childhood moderates the association between regulatory problems in infancy and trajectories of co-developing internalizing and externalizing symptoms across childhood. Using PP-LCGA, we identified four distinct groups of children who differed in their longitudinal patterns of co-developing internalizing and externalizing symptoms. Our findings showed that the association between infant regulatory problems and internalizing and externalizing symptom trajectories did not differ meaningfully based on the level of screen media exposure at 3 years. Rather, both regulatory problems at 9 months and excessive screen media exposure at 3 years, particularly screen media exposure of > 2 h per day, were associated with increased likelihood of internalizing and externalizing symptom trajectories across childhood.

To the best of our knowledge, this is the first study to report on the rate of infant regulatory problems in Ireland (22.4%), which is similar to the findings of studies from other countries [13]. Consistent with previous research [8, 28, 30], we identified a large (61.3%) normative class of internalizing and externalizing symptoms (i.e., low stable internalizing and low decreasing externalizing symptoms), and a small (6.3%) high-risk class (i.e., moderate increasing internalizing and high increasing externalizing symptoms), as well as two further classes with one large class characterized by stable externalizing symptoms (class 2; 20%) and the other one characterized by increasing internalizing symptoms (class 3; 12.4%).

The main finding of the current study was that the effect of regulatory problems at 9 months of age on the likelihood of internalizing and externalizing symptom trajectories does not depend on excessive screen media exposure at 3 years of age. Infant regulatory problems at 9 months of age are associated with an increased likelihood of all internalizing and externalizing symptoms trajectories beyond the impact of excessive screen media exposure at 3 years with the largest influence on the high-risk class. To illustrate, regulatory problems at 9 months increased the likelihood of the high-risk internalizing and externalizing symptoms trajectory by 36% with narrow confidence intervals indicating stability of the estimate. These findings are consistent with previous studies reporting associations between regulatory problems in infancy and increased likelihood of childhood internalizing and externalizing symptom trajectories with the largest effect on the severe and stable class [7, 8]. The current findings further support the notion that regulatory problems in infancy could serve an early phenotype for emotional and behavioural dysregulation, leading to the manifestation of co-occurring internalizing an externalizing symptoms across childhood [14, 17, 18]. Within the developmental cascades framework, it could be inferred that regulatory problems serve as a starting point of a trajectory of dysregulation across time and developmental domains [37]. Future research is required to explore other environmental determinants (such as peer bullying and harsh parenting) in understanding the outcomes of early regulatory problems.

Our findings showed that excessive screen media exposure in early childhood has an independent effect on internalizing and externalizing symptom trajectories beyond the impact of regulatory problems in infancy. Particularly, screen exposure of > 2 h was associated with increased likelihood of all trajectories with a large impact on the likelihood of high-risk internalizing and externalizing symptom trajectory (71% increase). This estimate had a wide confidence interval range which suggests that the precision is low and means that replication of the findings using other samples might not reveal a similar point-estimate. Screen media exposure for > 2 h had a smaller but more precise impact on classes 2 (moderate and stable externalizing symptoms, 26% increase) and 3 (increasing internalizing symptoms, 32% increase) with narrow confidence intervals. The association between excessive screen media exposure and class 3 was even evident when screen exposure was only > 1 h. This finding suggests that excessive screen media exposure in early childhood might have a particular influence on the development of internalizing symptoms, consistent with the findings of a previous study that investigated the bidirectional associations between screen exposure and internalizing and externalizing symptoms across childhood using the same cohort [24]. This association could be explained by the displacement of sleep, physical activity, and the duration of social interactions with parents and siblings. However, evidence supporting the displacement hypothesis is currently limited, with the impact of decreased physical activity being evident mainly for boys [38, 39]. Therefore, future studies are needed to systematically investigate the role of sleep, physical activity, and social interactions in the association between screen time and internalizing symptoms, considering possible sex differences.

Regarding clinical implications, these findings align with the theory of multifinality [40] and demonstrate that regulatory problems in infancy can lead to various maladaptive trajectories. Clinicians should recognize that regulatory problems starting in infancy could indicate early vulnerability, necessitating intervention to prevent a developmental cascade culminating in chronic self-regulatory problems and psychopathology. Offering parenting support concerning regulatory problems during the early years could mitigate the exacerbation of self-regulatory difficulties throughout childhood [41]. Additionally, clinicians need to understand the impact of excessive screen media exposure in early childhood on the development of psychopathology, particularly internalizing symptoms. This underscores the importance of discussing ‘screen media use’ limits with parents for children between 2 and 5 years of age as some parents may not be aware of such limits. Further, parents could also use the Family Media Plan as a resource to customize a plan [42].

The current study has several strengths including the prospective design from infancy to late childhood, repeated measurement of internalizing and externalizing symptoms across childhood, and a large sample size. However, it is important to acknowledge some limitations. First, all measurements were based on parental reports. While parent-reported measures are considered as less robust than objective measurements, they offer advantage of capturing behaviours that may not be observable during a short amount of time. Further, it is not feasible to include observational measures of regulatory problems (e.g., actigraphy, structured diaries), and screen time exposure in large-scale community and epidemiological investigations. Nonetheless, it is possible that using the same informant for the predictor and outcomes inflated the reported associations in the current study. Second, it is important to note the advancement of technology since the time when 3-year assessments were made in the current cohort. Thus, future studies are required to test whether similar findings are found in more recent cohorts. Third, in the current study, it was not possible to disentangle the influence of the level of parental engagement during screen exposure. Fourth, there was selective attrition with the participants who dropped out coming from a minority background and from families with low income. However, the retention rate was quite high, and the role of minority background and income was controlled in the analyses. Further, there is evidence that the dropout rates have little influence on the strength of prospective associations [43].

In conclusion, excessive screen media exposure in early childhood did not moderate the association between infant regulatory problems and childhood internalizing and externalizing symptoms. The current findings provide further support to the possibility that regulatory problems in infancy are the starting point of emotional and behavioural dysregulation across childhood. It further highlights the potential impact of screen time on children’s emotional and behavioural development.

## Electronic Supplementary Material

Below is the link to the electronic supplementary material.

Supplementary Material 1

## Data Availability

Sequence data that support the findings of this study are available through Irish Social Science Data Archive (https://www.ucd.ie/issda/) and the syntax files are available through Open Science Framework (https://osf.io/8rvmf/).

## Abbreviations

INT: Internalizing Symptoms
EXT: Externalizing Symptoms
WHO: World Health Organization (WHO)
AAP: The American Academy of Pediatrics
GUI: Growing Up in Ireland Study
