# Supplementary material for: Is the association between infant regulatory problems and trajectories of childhood co-developing internalizing and externalizing symptoms moderated by early screen media exposure?
Source: Eur Child Adolesc Psychiatry. 2024 Dec 31;34(7):2229–39. doi: 10.1007/s00787-024-02634-0 (PMC12334430; doi:10.1007/s00787-024-02634-0)
Supplement: Supplementary file 1 — Supplementary Material 1 [file 787_2024_2634_MOESM1_ESM.docx]

Supplementary Table 1. Bivariate correlations among variables

|  | 1 | 2 | 3 | 4 | 5 | 6 | 7 | 8 | 9 | 10 | 11 | 12 | 13 | 14 | 15 | 16 |
| --- | --- | --- | --- | --- | --- | --- | --- | --- | --- | --- | --- | --- | --- | --- | --- | --- |
| 1.Female | 1 |  |  |  |  |  |  |  |  |  |  |  |  |  |  |  |
| 2.Majority Ethnicity | -.01 | 1 |  |  |  |  |  |  |  |  |  |  |  |  |  |  |
| 3.Income | -.003 | .22** | 1 |  |  |  |  |  |  |  |  |  |  |  |  |  |
| 4.Second or later born | .03** | .05** | -.15** | 1 |  |  |  |  |  |  |  |  |  |  |  |  |
| 5. Gestational age in weeks | .01 | .006 | .02** | -.07* | 1 |  |  |  |  |  |  |  |  |  |  |  |
| 6. Married | .01 | .000 | .33** | .19** | -.03** | 1 |  |  |  |  |  |  |  |  |  |  |
| 7. Maternal psychological distress | -.02* | -.002 | -.14** | -.006 | -.02* | -.16** | 1 |  |  |  |  |  |  |  |  |  |
| 8. Regulatory problems | -.02* | -.08** | -.06** | .01 | .009 | -.02* | .14** | 1 |  |  |  |  |  |  |  |  |
| 9. Excessive screen media exposure | -.01 | -.11** | -.20** | .01 | -.02* | -.10* | .07** | .03** | 1 |  |  |  |  |  |  |  |
| 10. Internalizing symptoms at 3 years | -.04** | -.08** | -.12** | -.09** | -.04** | -.10** | .14** | .08** | .11** | 1 |  |  |  |  |  |  |
| 11. Internalizing symptoms at 5 years | -.02* | -.03** | -.10** | -.10** | -.05** | -.11** | .16** | .07** | .10** | .43** | 1 |  |  |  |  |  |
| 12. Internalizing symptoms at 7 years | -.03* | -.05** | -.14** | -.10** | -.03* | -.12** | .16** | .05** | .11** | .31** | .44** | 1 |  |  |  |  |
| 13. Internalizing symptoms at 9 years | -.01 | -.02* | -.11** | -.08** | -.03** | -.13** | .18** | .07** | .09** | .33** | .47** | .62** | 1 |  |  |  |
| 14. Externalizing symptoms at 3 years | -.09** | -.05** | -.14** | -.04** | -.01 | -.15** | .15** | .08** | .10** | .33** | .24** | .22** | .27** | 1 |  |  |
| 15. Externalizing symptoms at 5 years | -.14** | -.002 | -.12** | -.05** | -.03** | -.18** | .15** | .06** | .08** | .22** | .35** | .27** | .31** | .54** | 1 |  |
| 16. Externalizing symptoms at 7 years | -.18** | -.05** | -.13** | -.05** | -.01 | -.13** | .15** | .05** | .06** | .16** | .24** | .45** | .36** | .42** | .57** | 1 |
| 17. Externalizing symptoms at 9 years | -.18** | -.01 | -.12** | -.04** | -.03** | -.16** | .15** | .04** | .05** | .18** | .26** | .33** | .44** | .43** | .59** | .72** |

Supplementary Table 2. Unadjusted and adjusted odds of class membership for children with regulatory problems at 9 months and excessive screen media exposure at 3 years (either more than 1 or 2 hours) relative to children with regulatory problems at 9 months and no excessive screen media exposure at 3 years.

|  | Unadjusted | | | | Adjusted^a^ | | | |
| --- | --- | --- | --- | --- | --- | --- | --- | --- |
|  | Class1 | Class 2 | Class 3 | Class 4 | Class1 | Class 2 | Class 3 | Class 4 |
| Regulatory problems and excessive screen media exposure more than 1 hour vs Regulatory problems and no excessive screen media exposure | **.726 (.608-.866)** | **1.259 (1.014-1.562)** | **1.410 (1.091-1.823)** | .984 (.714-1.357) | .871 (.716-1.060) | 1.142 (.906-1.439) | 1.263 (.959-1.662) | .771 (.544-1.092) |
| Regulatory problems and excessive screen media exposure more than 2 hours vs Regulatory problems and no excessive screen media exposure | **.582 (.470-.722)** | **1.464 (1.134-1.888)** | **1.535 (1.136-2.074)** | 1.194 (.820-1.739) | **.756 (.591-.966)** | 1.283 (.968-1.701) | 1.224 (.875-1.712) | .888 (.583- 1.351) |

^a^Adjusted for female sex, ethnicity, maternal psychological distress, income, gestational age, parity, marital status

Class 1 (Low stable INT and low decreasing EXT); Class 2 (Low stable INT and moderate stable EXT); Class 3 (Moderate increasing INT and moderate decreasing EXT); Class 4 (Moderate increasing INT and high increasing EXT)
